# Efficacy of lenvatinib combined with TAS-102 as second-line therapy for advanced intrahepatic cholangiocarcinoma: a case report

**DOI:** 10.3389/fonc.2025.1695911

**Published:** 2025-12-04

**Authors:** Hailong Li, Zhiqiang Liu, Mei Zhang

**Affiliations:** 1 Graduate School, Anhui University of Chinese Medicine, Hefei, China; 2 Anhui University of Chinese Medicine, Hefei, China; 3 Oncology Department of Integrated Traditional Chinese and Western Medicine, The First Affiliated Hospital of Anhui Medical University, Hefei, China; 4 The Graduated School, Anhui Medical University, Hefei, China

**Keywords:** intrahepatic cholangiocarcinoma (ICC), lenvatinib (PubChem CID9823820), TAS-102 (trifluridine/tipiracil), TKI (tyrosine kinase inhibitor), PD-1 inhibitor resistance

## Abstract

Currently, chemotherapy remains the primary treatment modality for advanced intrahepatic cholangiocarcinoma (iCCA). However, the efficacy of existing regimens in patients requiring later-line therapy is limited, with low objective response rates and considerable adverse effects. Therefore, the development of effective and safe novel therapeutic strategies is of critical importance. We report a case of unresectable metastatic iCCA in which first-line therapy with a PD-1 inhibitor combined with a tyrosine kinase inhibitor (TKI) failed. The patient subsequently received second-line treatment with lenvatinib in combination with trifluridine/tipiracil (TAS-102), which resulted in significant tumor shrinkage and a partial response (PR) upon evaluation, without the occurrence of grade 3 or higher adverse events. The progression-free survival (PFS) was 13.33 months. This case suggests that lenvatinib combined with TAS-102 may demonstrate favorable antitumor activity and may represent a promising therapeutic option for advanced iCCA in the later-line setting.

## Introduction

Intrahepatic cholangiocarcinoma (iCCA) originates from the epithelial cells of the intrahepatic bile ducts and is a biliary tract carcinoma occurring above the secondary bile ducts in the liver. It is an aggressive tumor of the biliary system and accounts for 10–15% of all primary liver cancers (1). Surgical resection is the preferred treatment for iCCA; however, due to its insidious onset, most patients have lymph node or distant metastases at diagnosis and are not candidates for surgery (2). Furthermore, iCCA is prone to various forms of drug resistance during treatment and lacks effective therapies in advanced stages, resulting in poor prognosis (3). Therefore, exploring effective later-line treatment options for advanced iCCA is imperative.

For patients with unresectable and metastatic intrahepatic cholangiocarcinoma (iCCA), systemic chemotherapy remains the mainstay of treatment (4). Gemcitabine combined with cisplatin (Gem/CDDP) is considered the standard first-line regimen for advanced iCCA (5). Most subsequent-line treatments are based on 5-fluorouracil (5-FU)–containing regimens; however, their efficacy is often compromised by the development of secondary resistance, leading to a low objective response rate (ORR) and overall limited clinical benefit (6, 7). Trifluridine/tipiracil (TFT/TPI, TAS-102) is an oral chemotherapeutic agent distinguished by its antitumor activity even in tumors resistant to 5-FU (8). In addition, it exhibits highly selective inhibition of FGFR1–4, thereby impeding tumor proliferation and invasion (9).

Targeted therapy is playing an increasingly important role in the treatment of advanced intrahepatic cholangiocarcinoma (iCCA), primarily involving two categories of drugs: specific targeted agents and multi-target inhibitors. Due to the complex tumor microenvironment and cellular heterogeneity of iCCA, a series of targets mediating angiogenesis and metastatic invasion—such as VEGFR1-3, FGFR, PDGFRα, KIT, and RET—are excessively activated (10), which are key factors driving tumorigenesis and progression. Therefore, multi-target blockade agents have greater application prospects. Lenvatinib is an oral multi-target tyrosine kinase inhibitor that inhibits the expression of VEGF and FGFR through multiple pathways, thereby preventing tumor angiogenesis, metastasis, and progression (11). It has been approved for the treatment of unresectable hepatocellular carcinoma (HCC), indicating its potential activity against hepatobiliary system tumors. Furthermore, studies have found that multi-target tyrosine kinase inhibitors exhibit synergistic antitumor effects when combined with chemotherapy, which can enhance chemotherapy sensitivity, improve bioavailability, and reverse chemotherapy resistance (12). Therefore, we believe that lenvatinib combined with TAS-102 may have a synergistic effect, and clinical studies are being conducted.

**Figure 1:**
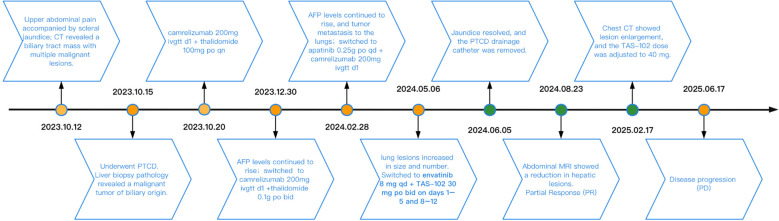
Timeline chart along with key dates for investigations and treatments of this patient.

In this report, we first obtained the patient’s consent to publish her clinical data before reviewing this case study. This was an elderly female patient with advanced iCCA (with liver and lung metastases) who achieved partial response (PR) after receiving second-line treatment with lenvatinib in combination with TAS-102. At the most recent follow-up time, progression-free survival (PFS) lasted approximately 13.33 months. **Figure 1** shows the patient’s diagnosis, treatment, and follow-up timeline.

## Case presentation

**Figure 2:**
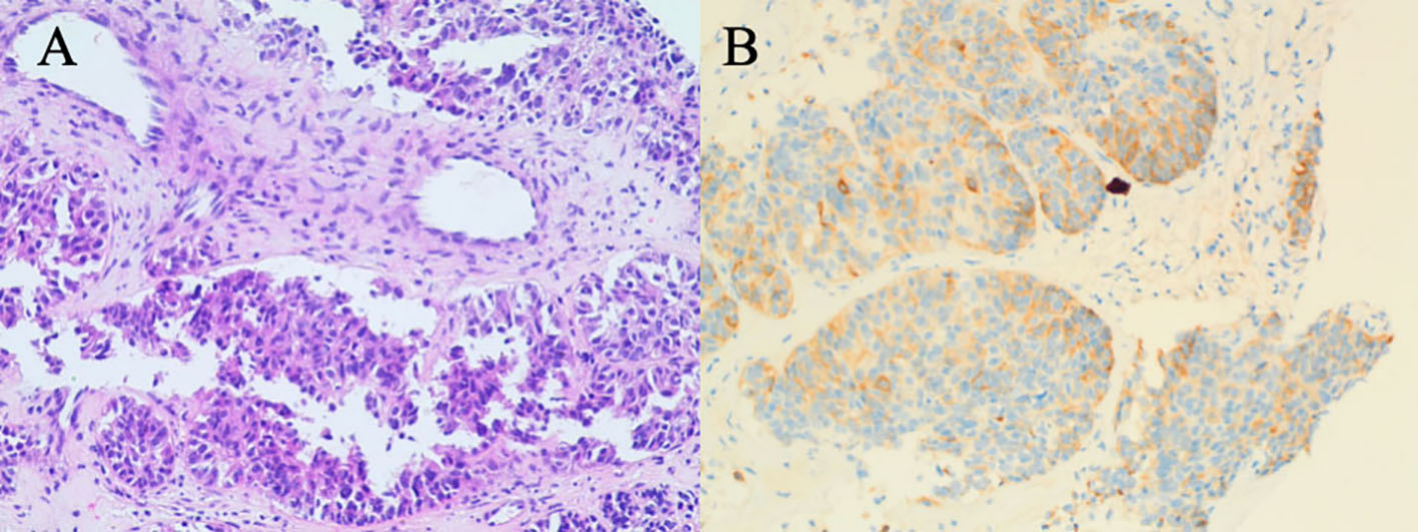
Liver biopsy pathology results on October 16, 2023. **(A)** H&E staining reveals sheets of epithelioid cells with irregular glandular arrangements, marked cellular atypia, and evidence of invasive growth, favoring a diagnosis of poorly differentiated adenocarcinoma. **(B)** The weak CK19 staining is suggestive of a biliary tract origin, and metastasis to the liver is a consideration.

On October 12, 2023, a 66-year-old woman with no history of smoking, alcohol consumption, hepatitis B virus infection, or family history of malignancy presented to a local hospital with a 5-day history of epigastric pain accompanied by mild scleral icterus. Abdominal ultrasonography of the hepatobiliary-pancreatic-splenic system revealed obstructive jaundice, with the level of obstruction located at the hepatic hilum and a space-occupying lesion in the upper segment of the common bile duct. Contrast-enhanced abdominal CT further demonstrated a mass in the upper segment of the common bile duct with involvement of the hepatic hilum and right hepatic duct, as well as a malignant hepatic lesion highly suggestive of metastasis. On October 15, 2023, upon presentation to our hospital, the patient exhibited marked jaundice of the skin and sclera, with tenderness in the right upper quadrant. Laboratory tests revealed elevated serum alpha-fetoprotein (AFP) at 2,364.68 ng/mL (reference range: 0–8.1 ng/mL). Liver function tests showed a total bilirubin (TBIL) level of 278.33 μmol/L (reference range: 0–23 μmol/L), direct bilirubin (DBIL) of 244.73 μmol/L (reference range: 0–4 μmol/L), and indirect bilirubin (IBIL) of 33.60 μmol/L (reference range: 0–19 μmol/L). On October 16, 2023, the patient underwent percutaneous transhepatic cholangial drainage (PTCD) involving the right anterior lobe and the left lateral inferior lobe bile ducts, along with a liver biopsy. Histopathological examination of the biopsy specimen (**Figure 2**) revealed poorly differentiated carcinoma within the hepatic tissue. Immunohistochemical staining showed the following profile: CK20 (−), CK7 (−), CK19 (weak+), GPC-3 (+), Hepatocyte (−), CDX2 (−), P40 (−), CgA (focally weak+), Syn (focally weak+), CD56 (−), TTF-1 (−), GATA-3 (−), and Ki-67 (20%+). These findings favored a biliary origin, although a metastatic source could not be excluded.

## Treatment and follow-up

After evaluation, the patient was deemed unsuitable for surgical intervention. Once the pathological diagnosis was established, and due to the patient’s refusal of intravenous chemotherapy, treatment was initiated on October 20, 2023, with two cycles of Camrelizumab 200 mg on day1 combined with thalidomide 100 mg orally at bedtime. On December 30, 2023, serum AFP increased to 4432.77 ng/mL. To control disease progression, the regimen was adjusted to Camrelizumab 200 mg on day1 combined with donafenib 0.1g orally twice daily for two cycles. On February 28, 2024, serum AFP further rose to 5258.58 ng/mL, and chest and abdominal CT revealed multiple metastases in both lungs and the liver. The treatment was subsequently switched to camrelizumab 200 mg on day1 combined with apatinib 0.25g orally once daily for two cycles.

**Figure 3:**
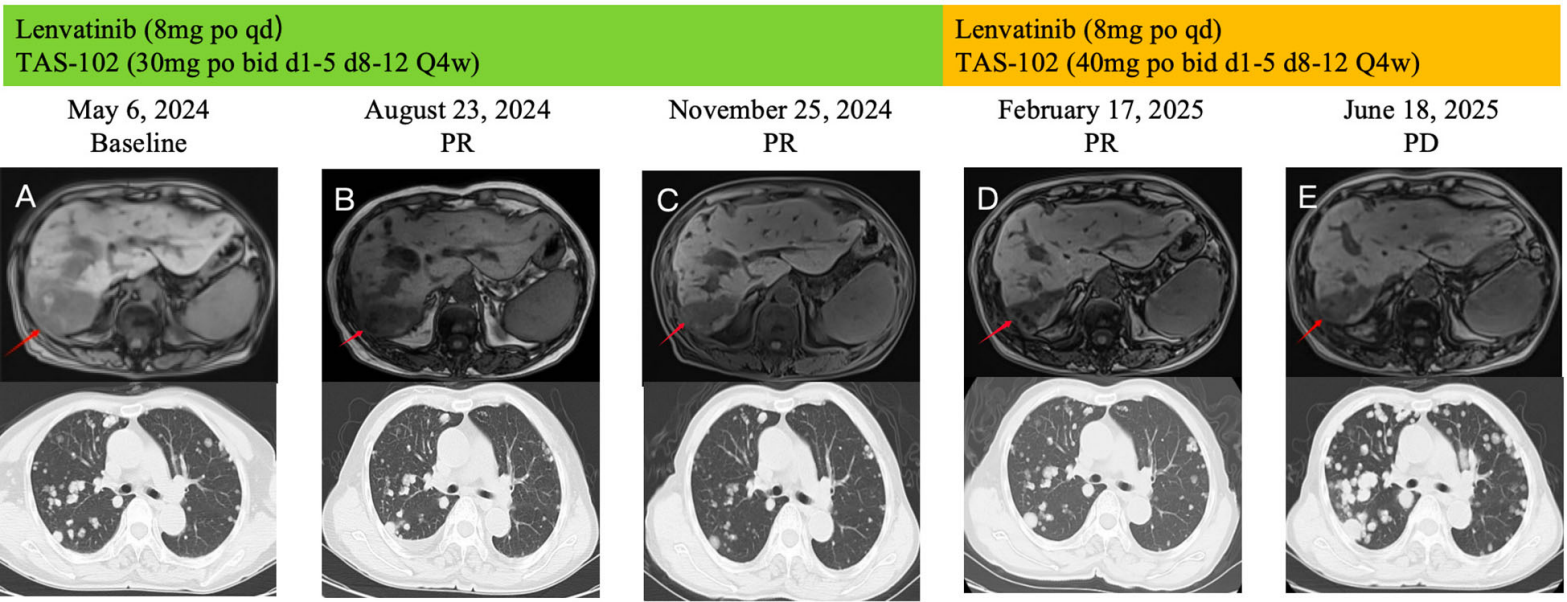
During the course of treatment, follow-up MRI was performed to assess changes in hepatic lesions **(A–D)** Gradually shrinks. **(D, E)** Gradually increase), and follow-up CT was performed to evaluate changes in pulmonary lesions **(A–C)** Gradually shrinks. **(C–E)** Gradually increase).

On May 6, 2024, the patient’s serum AFP continued to rise, reaching 11689.54 ng/mL. Abdominal MRI demonstrated enlargement of hepatic lesions, and chest CT revealed increased size and number of pulmonary lesions (**Figure 3A**). Treatment was initiated with lenvatinib 8mg orally once daily combined with trifluridine/tipiracil 30 mg orally twice daily on days 1-5 and 8-12 in 4-week cycles. After one cycle, the patient’s jaundice improved, total bilirubin returned to normal, and biliary drainage decreased. On June 5, 2024, the PTCD drainage catheter was removed at a local hospital. By August 23, 2024, serum AFP had decreased to 5258.58 ng/mL, and abdominal MRI demonstrated a significant reduction in hepatic lesions (**Figure 3B**). According to the Response Evaluation Criteria in Solid Tumors (RECIST 1.1) (13), the clinical response was assessed as a partial response (PR). The regimen was continued until November 25, 2024, when chest CT showed marked shrinkage of pulmonary metastases, with some lesions disappearing, and abdominal MRI revealed continued reduction of hepatic lesions (**Figure 3C**). Clinical response remained evaluable as PR. On February 17, 2025, the patient experienced chest tightness due to influenza. A chest CT scan at a local hospital showed enlarged lung lesions, while an abdominal MRI showed that the liver lesions were still shrinking (**Figure 3**). AFP levels rose to 4861.58 ng/ml. Considering that the patient’s recent weight gain had led to an increase in body surface area, the medication dosage was changed to lenvatinib 8mg po qd + trifluuridine tepiramycin 40mg po bid d1-5 d8-12 Q4w.Until June 18, 2025, the patient did not return for scheduled follow-up or dose adjustments. Chest CT at that time revealed marked enlargement and increased number of pulmonary lesions, while abdominal MRI showed slight enlargement of hepatic lesions (**Figure 3E**). Comprehensive assessment indicated disease progression (PD). Overall, the patient’s progression-free survival (PFS) was approximately 13.3 months. During treatment, the patient’s bilirubin levels dropped to the normal range, and even when the condition progressed, bilirubin levels did not rise. No treatment-related adverse reactions of grade 3 or higher occurred, and the ECOG score remained at 1-2 points. Overall, the treatment was well tolerated.

## Discussion

Chemotherapy remains a mainstay in the management of advanced intrahepatic cholangiocarcinoma (iCCA), with gemcitabine plus cisplatin (Gem/CDDP) established as the standard first-line regimen (14). For second-line treatment, chemotherapy regimens based on 5-fluorouracil (5-FU) are recommended, with FOLFOX (Oxaliplatin + 5-FU) being a representative example. Although it can significantly prolong overall survival (OS), its median progression-free survival (mPFS) is only 4.2 months, and there is still a risk of drug resistance during treatment leading to disease progression (7, 13, 15). Trifluridine/tipiracil (TFT/TPI, TAS-102) is an oral chemotherapeutic agent composed of trifluridine (TFT, which disrupts DNA synthesis) and tipiracil (TPI, which inhibits drug degradation). A key feature of TAS-102 is its ability to retain antitumor activity in tumors resistant to 5-FU. The cytotoxic effect of TFT primarily depends on the extensive incorporation of its metabolites into cellular DNA, inducing DNA dysfunction and subsequent cell death. In contrast, 5-FU inhibits thymidylate synthase (TS) to interfere with DNA biosynthesis. This difference in mechanisms of action helps explain the absence of cross-resistance between TAS-102 and 5-FU, allowing TAS-102 to circumvent resistance mechanisms associated with 5-FU (8). Furthermore, TAS-102 has been reported to exhibit highly selective inhibitory activity against FGFR1-4, thereby suppressing tumor proliferation and invasion (9). Currently, a small-sample clinical observation has preliminarily explored the efficacy of TAS-102 in CCA. As a second-line monotherapy for advanced CCA, the median progression-free survival (mPFS) reached 3.8 months. Its advantage lies in its effectiveness in patients who have previously received 5-FU treatment or are resistant to 5-FU (16).

With the rapid advances in molecular biology and genomics, an increasing number of therapeutic targets for malignant tumors have been identified, and targeted therapy has begun to play an increasingly important role in the treatment of advanced intrahepatic cholangiocarcinoma (iCCA). Targeted agents for iCCA can be broadly divided into two categories: specific target agents and multi-target inhibitors (17). Due to the complex tumor microenvironment and cellular heterogeneity of iCCA, specific target agents can only be used in selected patient subgroups, resulting in low population coverage and limited clinical applicability. In contrast, overactivation of multiple angiogenesis- and metastasis-related targets—such as VEGFR1–3, FGFR, PDGFRα, KIT, and RET—is common in iCCA (10) and represents a critical driver of tumorigenesis and progression, making multi-target blockade agents a promising therapeutic approach. Lenvatinib is an oral multi-target tyrosine kinase inhibitor that can inhibit VEGFR and FGFR through multiple pathways, thereby suppressing tumor angiogenesis, metastasis, and progression (11). It has been approved for the treatment of unresectable hepatocellular carcinoma (HCC), suggesting potential antitumor activity in hepatobiliary malignancies. Results of a phase II clinical trial involving 41 patients with advanced biliary tract cancer (CCA) treated as second-line or later showed that lenvatinib monotherapy achieved a disease control rate (DCR) of 78.0%, a median progression-free survival (mPFS) of 3.8 months, and a median overall survival (mOS) of 11.4 months. In contrast, regorafenib monotherapy, as a second-line treatment for biliary tract malignancies, had an mPFS of 3.0 months, indicating that lenvatinib could relatively prolong mPFS (18, 19), thus partially addressing the limited treatment options available in later lines.

Although combination regimens of immune checkpoint inhibitors (ICIs) and targeted agents are currently a research hotspot in advanced cholangiocarcinoma, the resistance rate to immune monotherapy or dual ICI therapy remains high (>60%), with only limited prolongation of progression-free survival (PFS) (20). In the present case, the patient experienced disease progression following multiple lines of ICI–targeted combinations, including PD-1 inhibitors plus tyrosine kinase inhibitors (TKIs) or immunomodulatory drugs (IMiDs), suggesting a typical immune-resistant tumor phenotype. This underscores the urgent need to explore novel therapeutic strategies that are not dependent on immune activation. The combination of lenvatinib and TAS-102 establishes a therapeutic framework integrating angiogenesis inhibition with cytotoxic chemotherapy, potentially overcoming the limitations of immunotherapy. Throughout treatment, no grade ≥3 toxicities were observed, and the patient’s Eastern Cooperative Oncology Group (ECOG) performance status remained at 1, indicating that this regimen possesses favorable tolerability. This feature is particularly advantageous for elderly or frail patients who are unable to tolerate intravenous chemotherapy.

Studies have found that anti-VEGF therapy can reduce tumor interstitial fluid pressure (IFP), thereby improving the penetration of chemotherapeutic drugs into the tumor (21). Anti-angiogenic drugs can also improve tumor blood flow structure and permeability through vascular normalization, which may promote drug delivery (22). Preclinical models have also shown that FGFR inhibitors can inhibit epithelial-mesenchymal transition and enhance DNA damage-induced apoptosis, which may enhance the cytotoxic effects of trifluorouridine incorporation (23). Therefore, it can be considered that lenvatinib reduces tumor vascular permeability and interstitial pressure by inhibiting VEGF and FGFR signaling pathways, thereby improving the intratumoral delivery of TAS-102 and enhancing its DNA damage-induced apoptosis. The combination of the two has a synergistic effect.

Based on this case report, we have preliminarily confirmed the effectiveness of lenvatinib combined with TAS-102. In the future, we will conduct systematic clinical studies to explore its mechanism of action, so as to further validate this combination regimen in prospective clinical studies. It is especially suitable for exploratory treatment of pan-drug resistant iCCA patients with PD-1 resistance and no FGFR fusion.

## Data Availability

The original contributions presented in the study are included in the article/supplementary material. Further inquiries can be directed to the corresponding author.
